# Surgeon practice variation drives disposable supply cost in inguinal hernia repair: a real-world health system analysis (2017–2025)

**DOI:** 10.1007/s00464-026-13200-x

**Published:** 2026-08-05

**Authors:** Ramsey M. Dallal, Sami Tannouri, Sultan S. Abdelhamid, Emery Cuellar, Francesco Palazzo

**Affiliations:** 1 Department of Surgery, Jefferson Health, 5501 Old York Rd, Suite 510, Philadelphia, PA 19141 USA; 2 Department of Surgery, Jefferson Health, Philadelphia, PA USA; 3 Sidney Kimmel Medical College, Thomas Jefferson University, Philadelphia, PA USA

**Keywords:** Inguinal hernia repair, Surgeon practice variation, Disposable supply cost, Robotic surgery, Laparoscopic surgery, Value-based surgery

## Abstract

**Background:**

As robotic platform adoption expands, differences in procedural cost for inguinal hernia repair are frequently attributed to surgical technology. However, the relative contribution of surgical approach versus surgeon-specific practice patterns to cost variation remains incompletely defined.

**Methods:**

We conducted a retrospective cohort study of adults undergoing inguinal hernia repair within a multi-hospital healthcare system from 2017 to 2025. Mixed-effects regression models evaluated direct disposable supply cost and operating room time, adjusting for patient characteristics, case complexity, surgeon experience, calendar year, and clustering at both surgeon and hospital levels.

**Results:**

The cohort included 14,313 repairs performed by 137 surgeons across 29 hospitals (open 43.8%, robotic 46.7%, laparoscopic 9.5%). Robotic utilization increased from 8.7 to 66.0% by 2024, largely replacing laparoscopy; 62% of surgeons performed only one approach. After adjustment, mean direct disposable supply cost was $567 for open, $1,527 for robotic, and $1,741 for laparoscopic repair (robot vs. laparoscopic: − $213, 95% CI − $274 to − $153, *p* < 0.001). Adjusted mean operating room time was 119 min for open, 142 for robotic, and 120 for laparoscopic repair (robotic vs. open: + 23.1 min, *p* < 0.001). Robotic repair was associated with lower odds of overnight admission (OR 0.73, 95% CI 0.55–0.96, *p* = 0.022). Within each platform, surgeon-level cost deviations ranged up to $777 (open) and $196 (robotic) above the platform mean, often exceeding between-platform differences. Standardized per 1,000 cases, combined potentially avoidable cost (supplies plus operating room time at $75/min) was $638,000 for robotic repairs.

**Conclusions:**

Surgeon-level practice variation is a major contributor to procedural cost variation in inguinal hernia repair and may provide an important target for value-improvement efforts. Because most surgeons in this cohort performed only one approach, such efforts are likely to have the greatest practical impact when directed at preference-card standardization and peer benchmarking within the platforms where surgeons already practice.

**Supplementary Information:**

The online version contains supplementary material available at https://doi.org/10.1007/s00464-026-13200-x.

Inguinal hernia repair is among the most commonly performed general surgery operations and represents a substantial source of procedural spending. Although open mesh repair remains widely utilized, minimally invasive approaches have expanded steadily, with randomized trials demonstrating reduced postoperative pain, faster recovery, and lower rates of chronic groin pain [1–3]. As these clinical benefits have become established, hernia research has shifted toward questions of cost, efficiency, and value.

Robotic inguinal hernia repair has been central to this debate. Early trials and national database analyses reported longer operative times and higher procedural costs for robotic repair compared with laparoscopy, without clear differences in short-term outcomes [4–6]. Meta-analyses have largely confirmed these findings [7–9], and much of the literature has framed cost differences primarily as a function of surgical technology. This platform-centric framing is likely incomplete. Propensity-matched studies show that cost differences vary more across surgeons and institutions than across platforms [10,11], and the RIVAL trial found no meaningful outcome differences between robotic and laparoscopic repair at two years [5].

Across multiple common operations, surgeon-level differences account for a substantial proportion of variation in disposable supply cost and operating room utilization, often exceeding the influence of patient characteristics or approach [12–14]. Yet studies of inguinal hernia repair have rarely disentangled platform effects from surgeon- and institution-level variation. Many prior analyses rely on charges rather than direct costs, pool minimally invasive approaches into a single category, or fail to account for clustering at both surgeon and hospital levels [15,16]. Moreover, few studies have examined whether robotic repair primarily substitutes for laparoscopy rather than expanding access to minimally invasive repair, an important consideration for interpreting cost comparisons over time [17].

Accordingly, the objective of this study was to determine how the surgical approach relates to direct disposable supply costs and operating room time across a large, multi-hospital cohort of inguinal hernia repairs, and to quantify the extent to which cost variation is attributable to individual surgeons’ practice patterns within their institutional settings.

## Materials and methods

### Study design and data sources

This retrospective cohort study included adult patients undergoing inguinal hernia repair within a large, integrated multi-hospital healthcare system between January 1, 2017, and December 31, 2025. The system encompasses academic medical centers and community-based hospitals across six geographic divisions. Data were derived from linked institutional sources, including the electronic health record, operating room scheduling systems, and enterprise financial cost-accounting databases.

The study used de-identified retrospective data and was deemed exempt by the institutional review board (iRISID-2025-0164). Reporting follows STROBE guidelines.

### Study population and surgical approach

All inguinal hernia repairs performed as the primary procedure during the study period were eligible. Surgical approach was classified as open, laparoscopic, or robotic based on operative documentation and standardized procedural coding. Cases with an ambiguous approach were excluded from multivariable analyses.

### Exposure and outcomes

Surgical approach (open, robotic, or laparoscopic) was the primary variable of interest, with open repair as the reference. Two outcomes were evaluated. Direct disposable supply cost per case was defined as institutionally captured direct costs attributable to disposable surgical supplies used during the index operation. This measure includes per-use charges for robotic instruments, which have a limited number of uses (typically approximately $200 per instrument, with three instruments per case) and are captured in the institutional cost-accounting system as a disposable supply expense. Implant-related costs, primarily mesh, were analyzed separately and excluded from the primary supply cost outcome.

The second primary outcome was operating room time, defined as the total time the room was occupied (“door-to-door” time). Length of stay was evaluated as a secondary outcome.

### Covariates and risk adjustment

Models were adjusted for patient demographics (age, sex), clinical factors (ASA class, case complexity defined by incarceration or strangulation, bilateral repair), and operational factors (admission class, hospital division, calendar year).

ASA class was recorded for 74% of patients (10,644 of 14,313) and body mass index for 57% (8150 of 14,313). We examined the pattern of this missingness and found that it was concentrated at the level of the contributing site rather than the patient or surgical approach: one division recorded no ASA values and two divisions recorded no BMI values in their source systems, and BMI capture also improved over the study period. This pattern is consistent with differences in electronic data capture across hospitals rather than informative, patient-level missingness. ASA class was retained as a covariate, so the primary models analyzed complete cases for this variable; because the division that recorded no ASA performed almost exclusively robotic repair, the complete-case cost sample underrepresents robotic cases relative to the full cohort. BMI was excluded from the primary models given its higher, site-structured missingness, and a prespecified sensitivity analysis restricted to patients with recorded BMI did not materially alter the estimates (Supplemental Table 2). Availability of ASA and BMI by division and by year, and a comparison of analytic and excluded cases are reported in Supplemental Table 6.

### Surgeon experience

Surgeon experience was approximated as each surgeon’s cumulative running case count during the study period for each approach (open, laparoscopic, robotic). These counts served as adjustments for experience accumulation over time rather than direct measures of individual learning curves, which would require a different study design. Experience gained before the study period could not be captured.

### Statistical analysis

To account for the fact that outcomes cluster within individual surgeons and hospitals, that is, a given surgeon’s cases tend to be more alike than cases across different surgeons, we used hierarchical regression models (mixed-effects linear regression) with separate adjustments for surgeon-to-surgeon and hospital-to-hospital differences. This approach isolates the effect of the surgical platform from the individual surgeon’s habitual practice pattern and institutional environment. The same covariate specification was used for both cost and operating room time. Overnight admission (≥ 1-day length of stay) was modeled using an analogous logistic regression.

To quantify surgeon-level variation, we generated surgeon-specific cost estimates (technically, best linear unbiased predictions or BLUPs), each representing how much a given surgeon’s supply cost deviates from the platform average after adjusting for patient and case factors. We then defined “potentially avoidable cost” as the difference between each surgeon’s estimated cost and that of the 25th percentile performer (a relatively efficient peer), summed across the cohort and standardized per 1000 cases. This construct illustrates the scale of opportunity for cost reduction rather than a prescriptive target; some variation reflects clinically appropriate responses to patient anatomy and intraoperative findings not captured by available data. Sensitivity analyses using median and zero benchmarks are presented in Supplemental Table 3. Operating room time was valued at $75 per minute, consistent with published estimates [18].

Pairwise comparisons between approaches were tested using standard statistical contrasts with 95% confidence intervals. Analyses used Stata 19.5 (StataCorp).

## Results

### Study cohort

A total of 14,313 inguinal hernia repairs were performed by 137 surgeons across 29 operative locations. Overall, 43.8% were open (*n* = 6,275), 46.7% robotic (*n* = 6,681), and 9.5% laparoscopic (*n* = 1,357). Baseline characteristics are summarized in Table 1.

**Table 1:** Baseline characteristics by surgical approach (*N* = 14,313)

Characteristic	Open (*n* = 6,275)	Robotic (*n* = 6,681)	Laparoscopic (*n* = 1,357)	*P*
Patient demographics				
Age (years), mean (SD)	61.6 (15.7)	57.7 (14.2)	56.4 (16.1)	< 0.001
Male sex, *N* (%)	5653 (90.1)	6093 (91.2)	1228 (90.5)	0.093
Clinical characteristics				
Bilateral repair, *N* (%)	150 (2.4)	953 (14.3)	287 (21.1)	< 0.001
Complex (strangulated), *N* (%)	123 (2.0)	13 (0.2)	20 (1.5)	< 0.001
Cohort summary				
Operative locations		29		
Unique surgeons		137		
Mean cases per surgeon		105		
Admission type (overall)	*N* (%)			
Outpatient elective	13,394 (93.6)			
Inpatient unscheduled	794 (5.5)			
Emergency	576 (4.0)			
Unadjusted outcomes, mean (SD)				
Direct disposable supply cost (USD)	$488 ($672)	$1590 ($577)	$1822 ($804)	< 0.001
Operating room time (min)	109 (43)	123 (43)	114 (45)	< 0.001
Implant cost (USD)	$208 ($472)	$263 ($360)	$278 ($319)	< 0.001

*P* values from Pearson chi-squared test (categorical) or Kruskal–Wallis test (continuous). Admission type and ASA classification reported for overall cohort; ASA data available for 74% of patients. *SD* standard deviation

### Temporal trends in surgical approach

**Fig. 1:**
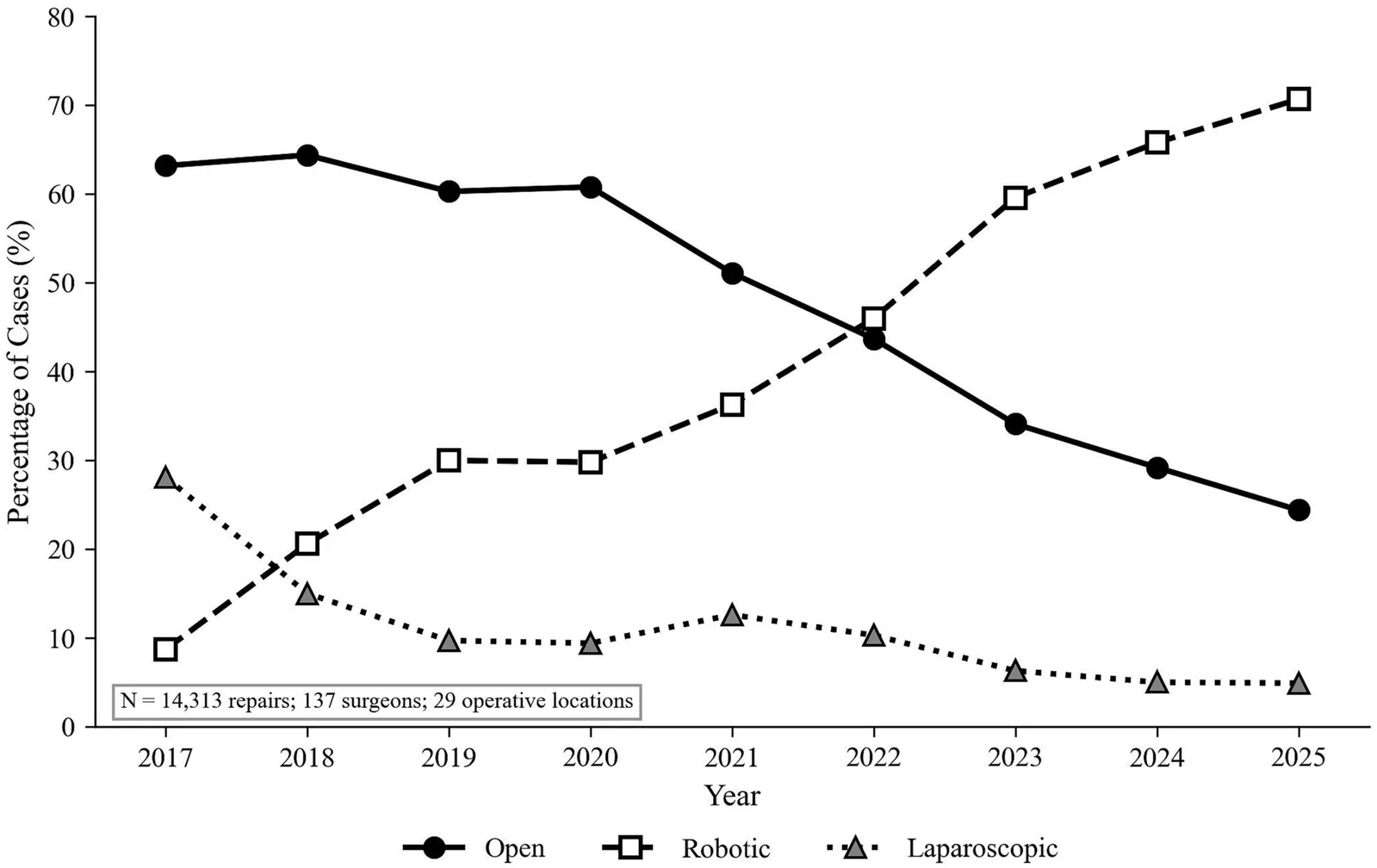
Temporal shift in surgical approach. Annual percentage of inguinal hernia repairs by approach, 2017–2025. *N* = 14,313 repairs; 137 surgeons; 29 operative locations

**Fig. 2:**
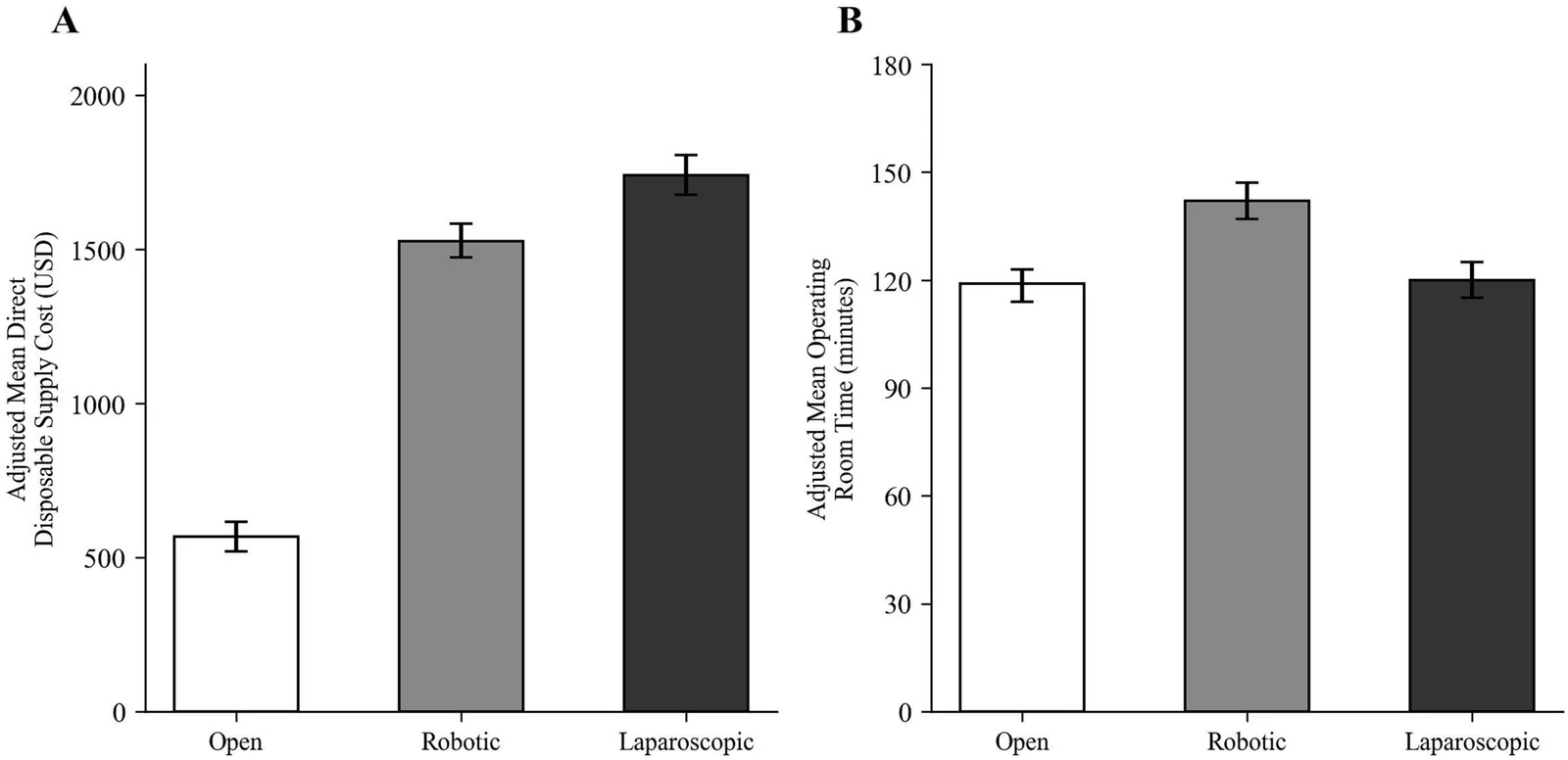
Cost-time trade-off across platforms. Adjusted mean direct disposable supply cost (**A**) and operating room time (**B**) by approach, with 95% confidence intervals. Derived from the mixed-effects models in Tables 2 and 3

Robotic utilization increased from 8.7 in 2017 to 66.0% by 2024, while laparoscopic repair declined from 28.1 to less than 5% (Fig. 1). Open repair declined modestly, from 63.2 to 29.2%. By 2022, robotic repair had surpassed open repair as the most common approach, consistent with robotic substitution for laparoscopic repair rather than displacement of open repair (Fig. 2).

**Table 2:** Mixed-effects linear regression for direct disposable supply cost per case (*N* = 9,925)

Variable	Coefficient (USD)	SE	*P*	95% CI
Surgical approach				
Open	Reference			
Robot	960	23	< 0.001	914–1006
Laparoscopic	1173	29	< 0.001	1116–1231
Bilateral repair				
Unilateral	Reference			
Bilateral	191	22	< 0.001	148–234
Surgeon experience				
Running laparoscopic count	0.75	0.22	0.001	0.32–1.18
Running robotic count	0.02	0.10	0.825	− 0.18 to 0.22
Running open count	0.06	0.09	0.539	− 0.12 to 0.23
Year of surgery	10.2	4.1	0.013	2.2–18.2
Case complexity				
Straightforward	Reference			
Complex (strangulated)	495	58	< 0.001	381 to 608
Sex				
Female	Reference			
Male	6.6	18.2	0.716	− 29 to 42
Age (per year)	− 0.13	0.37	0.718	− 0.85 to 0.59
ASA classification				
ASA 1	Reference			
ASA 2	32	26	0.223	− 19 to 82
ASA 3	31	28	0.275	− 25 to 86
ASA 4	39	47	0.397	− 52 to 130

Random Effects			
Component	Variance	SE	95% CI
Surgeon (intercept)	14,731	5786	6822 to 31,809
Hospital (intercept)	25,562	7232	14,682 to 44,505
Residual	413,831	5924	402,382 to 425,605

LR test vs. linear model: *χ*^2^(2) = 1028.1, *p* < 0.001

Robot vs. Laparoscopic contrast: − $213 (95% CI − $274 to − $153, *p* < 0.001)

Adjusted Marginal Means: Open $567 (95% CI $519–$616); Robot $1527 ($1473–$1582); Laparoscopic $1741 ($1676–$1806)

*ASA* American Society of Anesthesiologists, *SE* standard error, *CI* confidence interval, *LR* likelihood ratio.

Division fixed effects (Division A [reference], Division B, Division C, Division D, Division E, Division F) and admission class included in model but omitted from table for brevity.

**Table 3:** Mixed-effects linear regression for operating room time, door-to-door minutes (*N* = 10,644)

Variable	Coefficient (min)	SE	*P*	95% CI
Surgical approach				
Open	Reference			
Robot	23.1	1.25	< 0.001	20.7–25.6
Laparoscopic	0.83	1.55	0.593	− 2.2 to 3.9
Bilateral repair				
Unilateral	Reference			
Bilateral	27.1	1.19	< 0.001	24.8–29.4
Surgeon experience				
Running laparoscopic count	− 0.093	0.012	< 0.001	− 0.12 to − 0.07
Running robotic count	− 0.003	0.006	0.601	− 0.01 to 0.01
Running open count	0.002	0.005	0.718	− 0.01 to 0.01
Year of surgery	− 0.93	0.23	< 0.001	− 1.38 to − 0.48
Case complexity				
Straightforward	Reference			
Complex (strangulated)	38.0	3.0	< 0.001	32.0–43.9
Sex				
Female	Reference			
Male	8.5	1.2	< 0.001	6.2–10.9
Age (per year)	− 0.10	0.03	< 0.001	− 0.15 to − 0.05
ASA classification				
ASA 1	Reference			
ASA 2	5.8	1.4	< 0.001	3.1–8.5
ASA 3	9.5	1.5	< 0.001	6.5–12.5
ASA 4	9.9	2.5	< 0.001	4.9–14.8

Random effects			
Component	Variance	SE	95% CI
Surgeon (intercept)	247	77	134 to 454
Hospital (intercept)	199	49	123 to 323
Residual	1,249	17	1,215 to 1,283

LR test vs. linear model: *χ*^2^(2) = 249.6, *p* < 0.001

Robot vs. Laparoscopic contrast: 22.3 min (95% CI 19.0 to 25.5, *p* < 0.001)

Adjusted Marginal Means: Open 119 min (95% CI 114–123); Robot 142 min (137–147); Laparoscopic 120 min (115–125)

*ASA* American Society of Anesthesiologists, *SE* standard error, *CI* confidence interval, *LR* likelihood ratio.

Division fixed effects and admission class included in model but omitted from table for brevity.

The number of surgeons performing robotic repair increased from 6 in 2017 to 52 in 2025, while the number performing laparoscopic repair decreased from 22 to 12. Among the 137 surgeons, 85 (62%) performed only one approach (47 open-only, 35 robot-only, 3 laparoscopic-only), while 29 (21%) performed all three (Supplemental Table 1).

### Adjusted direct disposable supply cost

After adjusting for patient and case characteristics (Table 2; *n* = 9,925), robotic repair cost $960 more in disposable supplies than open repair (95% CI $914–$1,006, *p* < 0.001), and laparoscopic repair cost $1,173 more (95% CI $1,116–$1,231, *p* < 0.001). Notably, robotic repair was $213 less expensive than laparoscopic repair (95% CI − $274 to − $153, *p* < 0.001), a 12% difference that was both statistically significant and clinically meaningful.

Adjusted average supply costs were open $567, robotic $1527, and laparoscopic $1741. Case complexity (strangulation or incarceration) added $495 per case (*p* < 0.001), bilateral repair added $191 (*p* < 0.001), and each calendar year added $10 (*p* = 0.013), reflecting modest cost inflation over time. Among experience measures, the running laparoscopic case count was associated with slightly higher supply cost ($0.75 per additional case, *p* = 0.001), likely reflecting secular cost inflation correlated with experience accumulation rather than a true experience effect. Robotic and open experience showed no significant association.

Implant costs were similar across approaches (mean: open $208, robotic $263, laparoscopic $278), confirming that non-implant disposables (robotic instrument charges, trocars, energy devices, and access-related supplies) drove observed cost differences.

### Adjusted operating room time

Robotic repair took 23.1 additional minutes compared with open repair (95% CI 20.7–25.6, *p* < 0.001), while laparoscopic and open times were similar (0.8 min difference, *p* = 0.59). The robotic-versus-laparoscopic difference was 22.3 min (95% CI 19.0–25.5, *p* < 0.001). Adjusted average operating room times were open 119 min, robotic 142 min, laparoscopic 120 min (Table 3; *n* = 10,644).

Greater laparoscopic case volume was associated with modest reductions in operating room time (− 0.09 min per case, *p* < 0.001), whereas robotic and open case volumes showed no significant association [19].

### Surgeon- and hospital-level variation

**Fig. 3:**
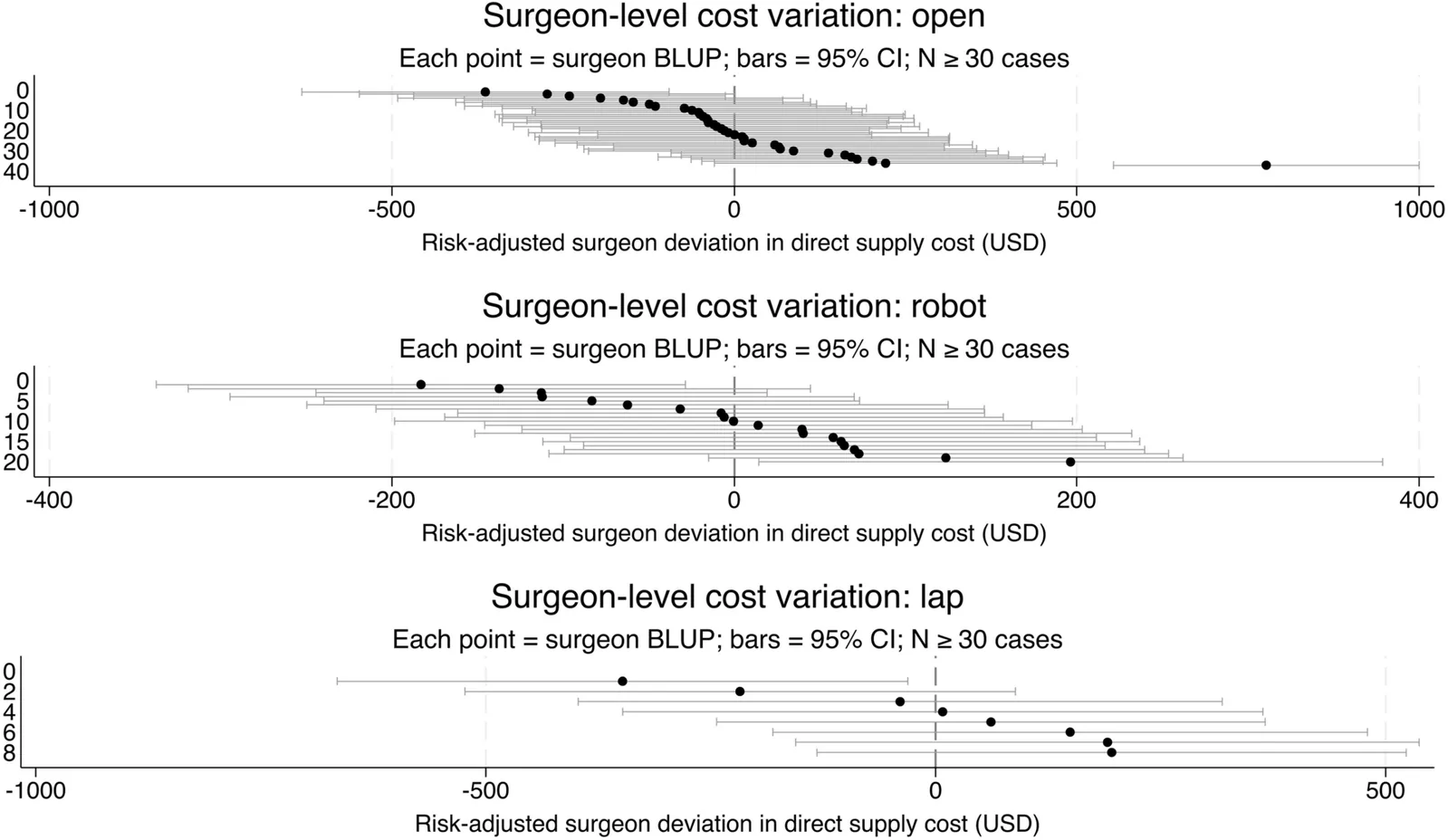
Surgeon-level cost variation by operative approach. Caterpillar plots of surgeon-specific BLUPs from approach-specific mixed-effects models, showing deviation in direct supply cost (USD) from the approach-specific mean. Bars indicate 95% CIs. Only surgeons with ≥ 30 cases shown (open: 37; robotic: 20; laparoscopic: 8)

Accounting for surgeon and hospital differences substantially improved the model (*p* < 0.001 for both cost and time), confirming that individual practice patterns matter. Figure 3 displays the surgeon-by-surgeon cost variation within each platform: among 37 open surgeons with 30 or more cases, adjusted cost deviations ranged from $363 below to $777 above the platform average; among 20 robotic surgeons, − $183 to + $196; and among 8 laparoscopic surgeons, − $348 to + $196. In other words, choosing the most versus the least efficient surgeon within a single platform could matter more than choosing between platforms.

### Potentially avoidable cost and operating room time

Standardized per 1000 cases, potentially avoidable disposable supply cost was $89,786 for open, $79,372 for robotic, and $131,873 for laparoscopic repairs. Potentially avoidable operating room time was 8828, 7446, and 4212 min, respectively. At $75 per minute, the combined avoidable cost (supplies plus time) was $751,865 per 1000 open cases, $637,830 per 1000 robotic cases, and $447,778 per 1000 laparoscopic cases. To put this in practical terms: a hospital performing 500 robotic inguinal hernia repairs per year could potentially save approximately $40,000 in supply costs and $279,000 in operating room time through surgeon-level practice optimization. Sensitivity analyses using more conservative benchmarks yielded lower estimates (Supplemental Table 3).

### Overnight admission

Patients undergoing robotic repair were less likely to stay overnight than those undergoing open repair (OR 0.73, 95% CI 0.55–0.96, *p* = 0.022). Laparoscopic repair showed a similar but nonsignificant trend (OR 0.78, *p* = 0.17).

## Discussion

The central finding of this study is that surgeon-level practice variation, rather than platform selection alone, is a major contributor to procedural cost in inguinal hernia repair and may represent an important target for value-improvement efforts. After adjusting for patient characteristics, case complexity, and institutional effects, individual surgeons’ supply cost deviations ranged up to $777 above the platform average for open repair and $196 for robotic repair. These within-platform differences, which reflect how individual surgeons select and use disposable supplies, often exceeded the differences between platforms themselves. This reframes the cost question from “which platform?” to “how is each surgeon using it?”

The cost-time trade-off between robotic and laparoscopic repair deserves emphasis. Robotic repair saved $213 in disposable supplies but required 22.3 additional minutes of operating room time compared with laparoscopy. At $75 per minute, this time difference amounts to approximately $1673 in operating room costs per case, far exceeding the $213 in supply savings. Disposable supply cost alone is therefore an incomplete measure of operative resource use. A full comparison of platforms requires accounting for total procedural cost, including staffing, anesthesia, capital depreciation, and downstream expenditures, data that were not available in this study. Robotic repair was also associated with a lower rate of overnight admission (OR 0.73 vs. open), which may partially offset time-related costs; however, this association should not be interpreted as a direct effect of the operative approach. Patients selected for open repair are frequently older or present with larger, incarcerated, or otherwise complex hernias that independently raise the likelihood of overnight admission and may make a minimally invasive approach less suitable. The lower overnight admission rate for robotic repair therefore likely reflects patient selection, and quantifying any genuine offset would require episode-of-care data.

Platform-level cost differences are otherwise consistent with prior trials and meta-analyses [4,6–9]. Implant costs were similar across approaches, confirming that non-implant disposables, including per-use robotic instrument charges (~ $600 per case for three instruments), trocars, energy devices, and access-related disposables such as balloon dissectors used in laparoscopic preperitoneal repair, drive observed cost differences. Our data did not distinguish between TEP and TAPP approaches, limiting direct evaluation of balloon dissector contributions.

The “potentially avoidable cost” framework requires careful interpretation: not all surgeon-level variation is unwarranted. Some reflects appropriate responses to patient anatomy, intraoperative findings, or unmeasured complexity such as hernia defect size, adhesion burden, or body habitus. In our sensitivity analysis (*n* = 8,150), mean BMI was similar across approaches (open 26.4, robotic 27.1, laparoscopic 26.5), and including BMI did not change primary findings (robot vs. lap: − $228, *p* < 0.001; Supplemental Table 2). The 25th percentile benchmark identifies the scale of opportunity; a more conservative median benchmark yields smaller estimates (1.8% of total robotic cost vs. 4.8% at the 25th percentile; Supplemental Table 3). The intent is to identify opportunities for investigation (preference-card review, peer benchmarking, and targeted observation) rather than prescribe a standard.

Actionable interventions begin with preference-card standardization: reviewing default supply lists and eliminating items routinely opened but not used. Prior studies estimate that 20–30% of disposable items opened during common general surgery procedures go unused [15]. Peer benchmarking provides surgeons with cost and time profiles relative to de-identified colleagues on the same platform, enabling identification of outlier practices. These team-based approaches (encompassing circulating nurse familiarity, instrument count sheet standardization, and anesthesia workflow alignment) have reduced intraoperative costs by 5–15% without adverse effects on outcomes [15,16].

The evolving pool of surgeons also has implications for interpreting cost estimates. By 2025, only 12 surgeons still performed laparoscopic repairs, with a median of 3 cases per year, a self-selected group of experienced practitioners whose efficiency may make laparoscopic cost estimates appear more favorable than they would in broader practice. Conversely, the expanding robotic pool (52 surgeons by 2025) includes surgeons at various stages of adoption; our case-count adjustment partially accounts for this, but cannot capture experience gained before 2017. Greater laparoscopic volume was associated with a modest reduction in time (− 0.09 min/case, *p* < 0.001), whereas no comparable reduction was observed for robotic volume [19]. This likely reflects greater maturity of laparoscopic workflows and team familiarity rather than a fundamental difference in how quickly surgeons learn each platform and should not be interpreted as evidence that robotic learning curves are absent. Characterizing individual learning curves would require skin-to-skin operative times and milestone-based progression data, which are beyond the scope of this analysis.

The temporal substitution of robotic for laparoscopic repair, from 8.7 in 2017 to 66.0% by 2024, mirrors national trends [17]. In our cohort, 62% of surgeons performed only one approach, consistent with surgical training that is increasingly platform-specific. A practical consequence is that efforts to reduce cost are more likely to succeed when directed at the platform a surgeon already uses than at moving surgeons between platforms. At the same time, the declining availability of surgeons able to perform minimally invasive repair without robotic assistance is itself a concern. The ability to perform open and laparoscopic inguinal hernia repair remains clinically important, particularly at sites without robotic access, and the narrowing of this skill set warrants further study from a surgical-education standpoint and attention at the level of training programs and the profession. We did not measure surgeon training, case assignment, or the reasons individual surgeons selected a given approach, and our data cannot establish what these surgeons would have done in the absence of a particular platform.

### Limitations

This study has several limitations. First, outcomes were limited to disposable supply cost and operating room time; capital costs, staffing costs, anesthesia costs, recurrence, patient-reported outcomes, and total episode-of-care expenditures were not included, so comprehensive value assessment is not possible. In particular, our cost-accounting data capture per-use robotic instrument charges as disposable supplies but do not capture the capital cost of the robotic platform itself, including newer per-procedure lease or pay-per-use pricing arrangements; the total cost of robotic repair is therefore understated relative to open and laparoscopic repair. Second, operating room time reflects total room occupancy (in-room to out-of-room) rather than skin-to-skin time. Because room-occupancy time is shaped by team, turnover, anesthesia, and scheduling factors that are not under the individual surgeon’s direct control, the operating-room-time component of cost is partly operational rather than strictly surgeon-specific, and the time-based portion of the avoidable-cost estimate should be interpreted with this in mind. Third, operating room time was valued at a single rate of $75 per minute. Operating room cost accounting is controversial and varies substantially among institutions, and this assumption underlies several of our dollar-denominated estimates; the combined avoidable-cost figures should therefore be read as order-of-magnitude estimates rather than precise values. Relatedly, our data do not allow fixed and variable supply costs to be fully separated, which further limits precise cost attribution. Fourth, we did not formally partition the total cost variance into surgeon, hospital, and platform components; our inferences about the relative contribution of surgeon-level variation are based on adjusted within-platform deviations and improvement in model fit rather than a formal variance-components decomposition. Fifth, residual confounding from surgeon self-selection into approaches, unmeasured case factors (defect size, body habitus), and subspecialization may influence findings; the lower overnight admission rate observed for robotic repair, in particular, may reflect preferential selection of older patients and larger or complex hernias for open repair rather than an effect of the approach. Sixth, ASA class and BMI were incompletely recorded (74% and 57% of cases, respectively), and this missingness was non-random, concentrated at specific divisions and, for BMI, in earlier study years rather than distributed by patient or approach; because the division that recorded no ASA performed almost exclusively robotic repair, the complete-case cost analysis underrepresents robotic cases, although a BMI sensitivity analysis did not materially change the platform contrast. Seventh, we could not distinguish laparoscopic TEP from TAPP approaches. Eighth, platform adoption was highly concentrated at certain divisions (one division performed exclusively open repairs, another almost exclusively robotic), and although division was included as a fixed effect, platform comparisons remain partly entangled with site-level adoption patterns, teaching intensity, and case ecosystems. Ninth, we did not include reoperations, readmissions, or postdischarge costs as outcomes; for elective inguinal hernia repair, these events occur at low rates, nearly all patients are managed as outpatients, and these downstream costs are unlikely to be the primary driver of procedural cost variation. Finally, this analysis does not establish clinical superiority of one minimally invasive platform over another.

## Conclusion

Surgeon-level practice variation is a major contributor to procedural cost variation in inguinal hernia repair, is present across all operative platforms, and may provide an important target for value-improvement efforts. When operating room time is valued alongside disposable supplies, the combined potentially avoidable cost is substantial, on the order of $638,000 per 1,000 robotic cases, although this figure depends on the assumed cost of operating room time. Because most surgeons perform only one approach, health systems may achieve the greatest practical impact by pursuing preference-card standardization, peer cost benchmarking, and team-based efficiency initiatives within the platforms where surgeons already practice.

## Supplementary Information

Below is the link to the electronic supplementary material.

Supplementary file1 (DOCX 32 kb)
