# Supplementary material for: Surgeon practice variation drives disposable supply cost in inguinal hernia repair: a real-world health system analysis (2017–2025)
Source: Surg Endosc. 2026 Aug 5;40(11):9531–40. doi: 10.1007/s00464-026-13200-x (PMC13755040; doi:10.1007/s00464-026-13200-x)
Supplement: Supplementary file 1 [file 464_2026_13200_MOESM1_ESM.docx]

**SUPPLEMENTAL MATERIALS**

*Surgeon Practice Variation Drives Disposable Supply Cost in Inguinal Hernia Repair: A Real-World Health System Analysis (2017–2025)*

**Supplemental Methods**

***Surgeon Specialization Classification***

To characterize platform adoption patterns, we classified each surgeon by the number of distinct approaches performed during the entire study period (2017–2025). A surgeon was classified as single-approach if all recorded cases used one approach (open, robotic, or laparoscopic), dual-approach if two approaches were used, and triple-approach if all three were used. Supplemental Table 1 reports the number of unique surgeons performing each approach by calendar year, and the overall specialization distribution is reported in the table footnote.

***Body Mass Index Sensitivity Analysis***

Body mass index (BMI) was available for 8,150 of 14,313 patients (57%) and was excluded from primary models to avoid selection bias from nonrandom missingness. To evaluate whether BMI confounded the primary findings, we re-estimated the mixed-effects cost model (identical specification to Table 2) restricted to patients with recorded BMI, both with and without BMI as an additional covariate. We compared the robotic and laparoscopic coefficients and the robot-versus-laparoscopic contrast between the BMI-adjusted and primary models. Results are reported in Supplemental Table 2.

***Benchmark Sensitivity Analysis for Potentially Avoidable Cost***

The primary analysis benchmarked surgeon-level best linear unbiased predictions (BLUPs) against the 25th percentile performer within each approach. To assess the sensitivity of these estimates to benchmark choice, we repeated the avoidable cost calculation using two alternative benchmarks: (1) the median performer (50th percentile) and (2) zero (the grand mean, representing the average surgeon). For each benchmark, we summed the positive deviations above the benchmark across all surgeons with ≥30 cases and expressed the total as a percentage of the observed total disposable supply cost. Results for robotic cases are reported in Supplemental Table 3. The 25th percentile benchmark identifies the upper bound of opportunity; the median and zero benchmarks provide progressively more conservative estimates.

***Institutional Division Analysis***

The health system encompasses six geographic divisions, each with distinct institutional histories, trainee involvement, and patterns of surgical technology adoption. Supplemental Table 4 reports the distribution of surgical approaches across divisions. Division was included as a fixed effect in all primary models to account for systematic differences in case mix, cost structures, and institutional practice patterns. Notably, platform adoption was highly concentrated at certain divisions: Hospital 3 performed exclusively open repairs, while Hospital 6 performed almost exclusively robotic repairs. This near-complete segregation means that platform comparisons in the primary models are partly informed by between-division differences, despite the inclusion of division fixed effects. This limitation is acknowledged in the main manuscript.

***Combined Avoidable Cost and Operating Room Time Estimation***

To estimate the combined economic impact of surgeon-level variation, we calculated potentially avoidable operating room time using the same BLUP-based framework applied to the operating room time model (Table 3). Surgeon-level time deviations were benchmarked against the 25th percentile performer within each approach, and positive deviations were summed across surgeons with ≥30 cases. Operating room time was valued at $75 per minute, consistent with published estimates for mixed academic and community hospital settings [18]. This rate reflects operating room staffing, overhead, and opportunity costs but does not include anesthesia professional fees, capital depreciation, or post-anesthesia care unit costs. The combined avoidable cost (disposable supplies plus operating room time) was standardized per 1,000 cases for each approach and is reported in Supplemental Table 5. The hospital-scale example in the main text (500 robotic cases = ~$40,000 in supplies + ~$279,000 in time) is based on these estimates.

***Missing Data Pattern Analysis***

ASA class (recorded for 10,644 of 14,313 patients, 74%) and body mass index (8,150 of 14,313, 57%) were the two covariates with appreciable missingness. To determine whether these data were missing in an informative, patient-related pattern, we cross-tabulated the availability of each variable by surgical approach, by institutional division, and by calendar year, and compared the characteristics of cases included in the complete-case cost model with those excluded. ASA missingness was almost entirely a site-level phenomenon, and BMI missingness was structured by both site and calendar year; neither tracked patient- or approach-level clinical characteristics. Results are reported in Supplemental Table 6.

**Supplemental Tables**

**Supplemental Table 1.** Number of Unique Surgeons Performing Each Approach, by Year

The number of surgeons performing robotic repairs increased steadily from 6 in 2017 to 52 in 2025, while the number of surgeons performing laparoscopic repairs declined from 22 to 12. Total surgeon counts exceed the sum of individual approaches because some surgeons performed multiple approaches within a given year.

| **Year** | **Open** | **Robot** | **Lap** | **Total** |
| --- | --- | --- | --- | --- |
| 2017 | 37 | 6 | 22 | 38 |
| 2018 | 42 | 11 | 20 | 45 |
| 2019 | 44 | 12 | 18 | 46 |
| 2020 | 48 | 15 | 15 | 50 |
| 2021 | 63 | 31 | 29 | 80 |
| 2022 | 59 | 35 | 30 | 82 |
| 2023 | 64 | 44 | 24 | 85 |
| 2024 | 59 | 49 | 18 | 86 |
| 2025 | 48 | 52 | 12 | 80 |

Surgeon specialization (entire study period): 85 surgeons (62%) performed only one approach (47 open-only, 35 robot-only, 3 laparoscopic-only); 30 (22%) performed 2 approaches; 29 (21%) performed all 3. Total column reflects unique surgeons (a surgeon performing multiple approaches is counted once).

**Supplemental Table 2.** BMI Sensitivity Analysis: Mixed-Effects Cost Model Restricted to Patients with Recorded BMI (N = 8,150)

Mean BMI was similar across approaches (open 26.4, robotic 27.1, laparoscopic 26.5), and including BMI as a covariate did not materially alter the primary findings. The robot-versus-laparoscopic contrast changed from −$213 in the primary model to −$228 in the BMI-adjusted model, with BMI itself contributing $11.1 per unit increase (p<0.001).

|  | **Open** | **Robotic** | **Laparoscopic** |
| --- | --- | --- | --- |
| BMI, mean (SD) | 26.4 (4.7) | 27.1 (4.6) | 26.5 (4.5) |

Cost model with BMI (restricted to non-missing BMI):

Robot coefficient: $958 (SE 25, p<0.001)

Laparoscopic coefficient: $1,187 (SE 32, p<0.001)

Robot vs. Laparoscopic contrast: −$228 (95% CI −$296 to −$161, p<0.001)

BMI coefficient: $11.1 per unit (SE 1.6, p<0.001)

Primary model (N=9,925): Robot vs. Laparoscopic contrast: −$213 (95% CI −$274 to −$153, p<0.001).

Inclusion of BMI did not materially alter approach coefficients or the primary contrast.

**Supplemental Table 3.** Sensitivity Analysis: Potentially Avoidable Disposable Supply Cost Under Different Benchmarks (Robotic Cases, N = 20 Surgeons with ≥30 Cases)

The magnitude of potentially avoidable cost depends on the benchmark used to define “expected” performance. The 25th percentile benchmark (used in the primary analysis) represents a high-performance standard and yields the largest estimate. The median benchmark identifies the portion of variation above the typical surgeon, while the zero benchmark (grand mean) captures variation above the average. These sensitivity analyses demonstrate that the conclusion—surgeon-level variation represents a meaningful source of potentially avoidable cost—is robust to benchmark choice, though the absolute magnitude varies.

| **Benchmark** | **Avoidable Cost** | **% of Total** |
| --- | --- | --- |
| 25th percentile | $270,659 | 4.8% |
| Median | $102,700 | 1.8% |
| Zero (grand mean) | $113,068 | 2.0% |

Total observed robotic disposable supply cost: $5,642,375. Restricted to 20 surgeons with ≥30 robotic cases.

The 25th percentile benchmark identifies the upper bound of opportunity. The median and zero benchmarks provide more conservative estimates of the variation amenable to intervention.

**Supplemental Table 4.** Surgical Approach Distribution by Institutional Division

Platform adoption varied substantially across the six health system divisions. Hospital 3 performed exclusively open repairs, consistent with a community hospital lacking robotic infrastructure during the study period. Hospital 6 performed almost exclusively robotic repairs, reflecting early and comprehensive adoption of robotics. Hospital 2 had the highest laparoscopic utilization (21.0%), consistent with its role as the primary academic campus with established laparoscopic training programs. These patterns illustrate why division was included as a fixed effect in all primary models and why platform comparisons should be interpreted with awareness of this site-level confounding.

| **Division** | **Open N (%)** | **Robot N (%)** | **Lap N (%)** | **Total** |
| --- | --- | --- | --- | --- |
| Hospital 1 | 1,138 (66.9) | 499 (29.4) | 63 (3.7) | 1,700 |
| Hospital 2 | 1,922 (51.2) | 1,044 (27.8) | 790 (21.0) | 3,756 |
| Hospital 3 | 1,202 (100.0) | 0 (0.0) | 0 (0.0) | 1,202 |
| Hospital 4 | 1,122 (41.8) | 1,392 (51.8) | 173 (6.4) | 2,687 |
| Hospital 5 | 891 (36.6) | 1,214 (49.9) | 330 (13.6) | 2,435 |
| Hospital 6 | 0 (0.0) | 2,532 (100.0) | 1 (0.0) | 2,533 |

Division was included as a fixed effect in all primary models. Hospital was included as a random intercept. The near-complete segregation of platforms at Hospital 3 (open-only) and Hospital 6 (robotic-only) means that platform comparisons are partly entangled with site-level differences in case mix, cost structures, and practice patterns.

**Supplemental Table 5.** Potentially Avoidable Cost and Operating Room Time, Standardized Per 1,000 Cases

Combined avoidable cost includes both potentially avoidable disposable supply cost and potentially avoidable operating room time, valued at $75 per minute. For robotic repair, the combined avoidable cost of approximately $638,000 per 1,000 cases is driven primarily by operating room time ($558,000) rather than disposable supplies ($79,000). This illustrates that interventions targeting efficiency—such as team training, standardized workflows, and anesthesia coordination—may yield greater savings than supply cost reduction alone. For a hospital performing 500 robotic inguinal hernia repairs annually, these estimates correspond to approximately $40,000 in potentially avoidable supply cost and $279,000 in potentially avoidable operating room time.

| **Approach** | **N Cases** | **N Surgeons** | **Avoidable Supply Cost** | **Avoidable OR Minutes** | **Avoidable OR Time Cost ($75/min)** | **Combined Avoidable Cost** |
| --- | --- | --- | --- | --- | --- | --- |
| Open | 4,677 | 37 | $89,786 | 8,828 | $662,078 | $751,865 |
| Robot | 3,410 | 20 | $79,372 | 7,446 | $558,457 | $637,830 |
| Lap | 816 | 8 | $131,873 | 4,212 | $315,905 | $447,778 |

Surgeon-level BLUPs benchmarked to 25th percentile performer within each approach. Restricted to surgeons with ≥30 cases. Values standardized per 1,000 cases.

Not all surgeon-level variation is unwarranted; some reflects clinically appropriate responses to patient anatomy, intraoperative findings, or unmeasured case complexity. These estimates represent the upper bound of opportunity for investigation, not a prescriptive target.

OR time valued at $75/minute, consistent with Childers & Maggard-Gibbons [18].

**Supplemental Table 6.** Availability of ASA Class and Body Mass Index by Institutional Division

Recording of ASA class and BMI varied markedly across divisions and reflected a site-level data-capture characteristic rather than a patient- or approach-level phenomenon. One division (Hospital 6), which performed almost exclusively robotic repair, recorded no ASA values, and two divisions (Hospitals 3 and 6) recorded no BMI values.

| **Division** | **Total N** | **ASA recorded, n (%)** | **BMI recorded, n (%)** |
| --- | --- | --- | --- |
| Hospital 1 | 1,700 | 1,700 (100.0) | 1,673 (98.4) |
| Hospital 2 | 3,756 | 3,756 (100.0) | 3,441 (91.6) |
| Hospital 3 | 1,202 | 1,202 (100.0) | 0 (0.0) |
| Hospital 4 | 2,687 | 1,551 (57.7) | 1,495 (55.6) |
| Hospital 5 | 2,435 | 2,435 (100.0) | 1,541 (63.3) |
| Hospital 6 | 2,533 | 0 (0.0) | 0 (0.0) |
| Total | 14,313 | 10,644 (74.4) | 8,150 (56.9) |

Availability also varied by calendar year for BMI, which rose from 34.1% in 2017 to 59.6% in 2025, consistent with maturation of structured electronic documentation; ASA availability showed no consistent temporal trend (range 65.9% to 86.7% per year).

Because the predominantly robotic division recorded no ASA, the complete-case cost analysis (n = 9,925) under-represents robotic cases relative to the full cohort (robotic 35.7% of the analytic sample vs 46.7% overall) and over-represents open cases (52.9% vs 43.8%). Analytic and excluded cases were otherwise similar in sex (90.4% vs 91.2% male) and differed modestly in mean age (60.3 vs 57.0 years) and in the proportion of complex, incarcerated or strangulated, cases (1.4% vs 0.3%). Including BMI did not materially change the robotic-versus-laparoscopic cost contrast (Supplemental Table 2).

Hospital labels correspond to those in Supplemental Table 4. ASA, American Society of Anesthesiologists physical status classification; BMI, body mass index.

**Supplemental References**

18. Childers CP, Maggard-Gibbons M (2018) Understanding costs of care in the operating room. JAMA Surg 153(4):e176233
